# Effects of high and low 17β-estradiol doses on focal cerebral ischemia: negative results

**DOI:** 10.1038/srep03111

**Published:** 2013-11-01

**Authors:** Jakob O. Strom, Edvin Ingberg, Elvar Theodorsson, Annette Theodorsson

**Affiliations:** 1 Clinical Chemistry, Department of Clinical and Experimental Medicine, Faculty of Health Sciences, Linköping University, Department of Clinical Chemistry, County Council of Östergötland, Linköping, Sweden; 2 Neurosurgery, Department of Clinical and Experimental Medicine, Faculty of Health Sciences, Linköping University, Department of Neurosurgery, County Council of Östergötland, Linköping, Sweden

## Abstract

The reasons why some animal studies indicate that estrogens increase focal cerebral ischemic damage while others show estrogen-induced neuroprotection has hitherto not been fully elucidated. Recent evidence indicates that discrepancies in hormone administration paradigms, resulting in highly different serum hormone concentrations, may account for the dichotomy. The current study aimed to test this hypothesis. Sixty ovariectomized female rats were randomized into three groups differing in 17β-estradiol regimens, and transient focal cerebral ischemia was subsequently induced. All animals were subjected to a small functional testing battery, and three days after MCAo they were sacrificed for infarct size assessment. Infarct sizes did not differ between groups, however clear discrepancies were seen in body weight and feeding behavior. In comparison to sham-operated animals, ovariectomized rats rapidly increased in body weight, whereas the opposite was seen in rats receiving 17beta-estradiol. The weight gain in the ovariectomized rats was paralleled by an increased food intake.

Estrogens constitute a family of female sexual steroid hormones that exert widespread and profound physiological effects, and are consumed as menopausal hormone therapy and for contraception. The observation that premenopausal women suffer less from cerebral ischemia than men do, and that this difference diminishes during menopause^1^, has sparked intense research efforts to investigate the potential neuroprotective effects of the estrogens. Unfortunately, despite massive scientific investments, there is still a lack of consensus regarding the fundamental question of whether estrogens are neuroprotective^2^,^3^ or neurodamaging^4^,^5^.

In many of the animal studies investigating the effects of estrogens on ischemic stroke a middle cerebral artery occlusion model has been adopted. Although the variations are almost infinite, the common denominator for these methods is that the middle cerebral artery (MCA), which in humans is the most common vessel to be compromised during ischemic stroke, is occluded. The most frequently used method for occluding the MCA, called the “intraluminal filament method”, is accomplished by inserting a filament via the common carotid artery (CCA) in the throat region, and can be modified to produce either a transient or permanent obstruction of blood flow^6^. This method, adopted in the current article in its transient form, has previously yielded results indicating both protective^7^,^8^ and damaging^5^ effects of estrogens.

One of the rat studies in which 17β-estradiol, the most potent of naturally occurring estrogens, was found to contribute to neurodamage was performed in our laboratory. This finding was at that point in time highly unexpected, and prompted a thorough assessment of different methodological aspects including the estrogen administration methods used. The three most well-used administration methods for 17β-estradiol to rats were therefore investigated, finding that the commercially available slow-release pellets (from the company Innovative Research of America) used produced exceptionally high serum levels of the hormone^9^,^10^. Interestingly, these specific pellets had not only been used in our lab, but in all rat studies where estrogens had been reported neurotoxic, and the higher the 17β-estradiol dose in the pellets, the likelier the pellets were to augment the ischemic damage, which was shown in a meta-analysis^11^. Our initial estrogen-stroke experiment was therefore re-performed, with the only change that low-dose silastic capsules, that had earlier been reported to be neuroprotective, were used instead of the pellets. It was found that this switch of administration mode rendered 17β-estradiol protective instead of damaging^12^, thus confirming the pattern demonstrated in the meta-analysis^11^.

However, that one administration method, merely depending on the dose of estrogen delivered can be either neurotoxic or neuroprotective, remains to be proven. The aim of the current study was therefore to test whether a high-dose capsule (designed to produce approximately the same serum levels of 17β-estradiol as the earlier used neurotoxic pellets) would cause increased ischemic damage, while on the contrary a low-dose capsule would be neuroprotective. A possible mechanism for high-dose estrogens' detrimental effects is preoperative weight loss, which was why the secondary aim of the study was to assess the impact of estrogen treatments on feeding behavior and body weight.

## Results

### Lesion sizes, mortality and functional tests

**Figure 1:**
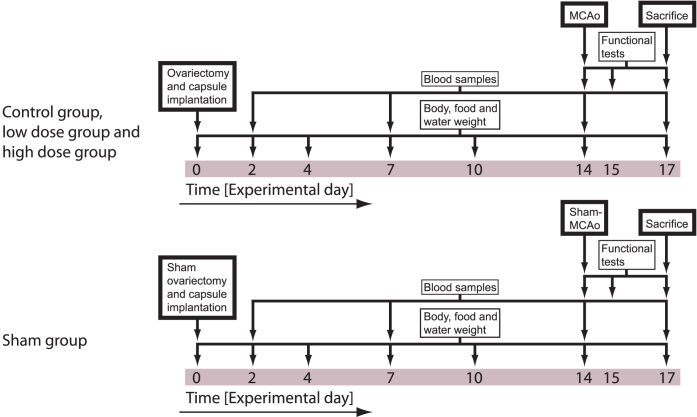
Overview of the experimental design. After ovariectomy/sham surgery and capsule implantation day 0, rats were subjected to blood sampling and weighing procedures. Day 14, all animals except the sham group were inflicted cerebral ischemia via MCAo. Functional tests (tail swing test and cylinder test) were performed prior to MCAo and on day 15 and 17. After the testing on day 17, animals were decapitated and infarct sizes analyzed. The rats in the sham group were subjected to the same protocol, except that ovariectomy and MCAo were sham procedures.

**Figure 2:**
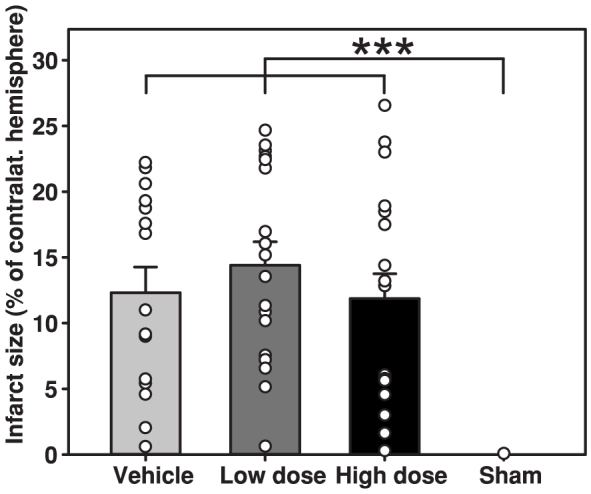
Infarct sizes (±SEM) in all groups. The white dots represent individual infarct sizes. There were no significant differences between the vehicle, low dose and high dose groups.

**Figure 3:**
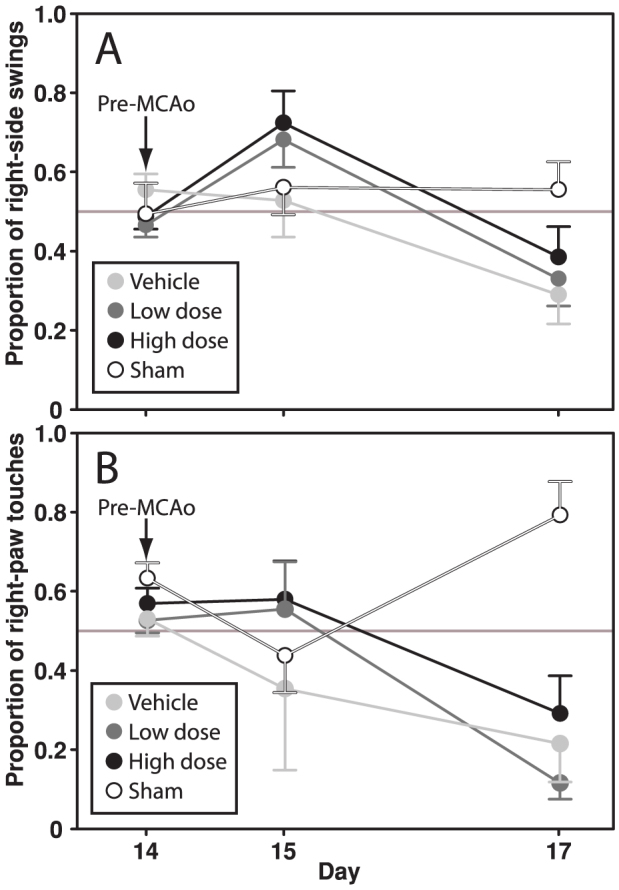
Tail swing (A) and cylinder (B) test performance (±SEM) prior to MCAo and on day 15 and just before sacrifice on day 17. No differences were seen between the vehicle, low dose and high dose groups on day 17. However, the sham group differed significantly from the other groups in cylinder test performance on day 17 (p < 0.001).

Sixty ovariectomized female rats were randomized into three groups differing in 17β-estradiol regimens; vehicle, low dose (180 μg/mL in one 3 cm long silastic capsule) and high dose (50 000 μg/mL in two 3 cm long silastic capsules). Following two weeks of treatment, left-side focal cerebral ischemia was induced via a transient intraluminal filament middle cerebral artery occlusion (MCAo) method. All animals were subjected to a small functional testing battery (cylinder test and tail swing test), and three days after MCAo they were sacrificed for infarct size assessment (see experiment overview in Figure 1). Except for the sham group, neither size of ischemic lesions nor functional test performance differed between groups (p > 0.05; Figure 2 and 3). The sham group differed significantly from the other groups in ischemic lesion size (p = 0.001; Figure 2) and cylinder test performance at day 17 (p < 0.001; Figure 3B).

The overall mortality was 13% (Table 1). If mortality was included in the statistical analysis of infarct sizes in a “total outcome” non-parametric model according to Kruskal-Wallis, still no differences were seen between the vehicle, low dose and high dose groups (p > 0.05).

### Body weights

**Figure 4:**
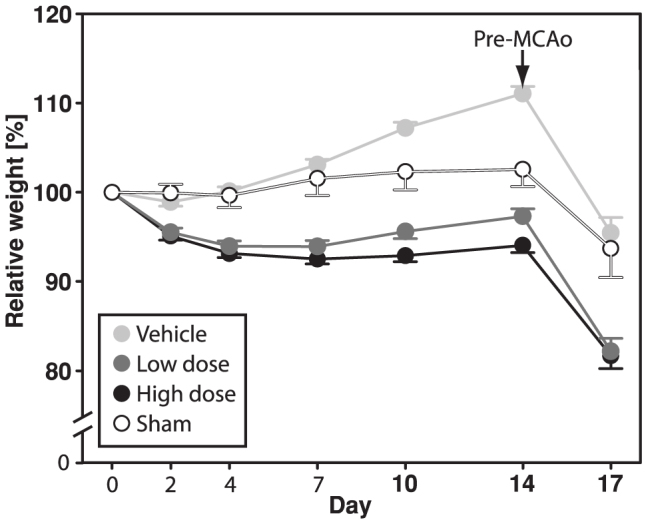
Body weights (presented normalized to each animal's body weight on day 0 ± SEM) were affected by ovariectomy and 17β-estradiol treatments. In the two-way ANOVA model, all groups differed significantly (p < 0.001 to p = 0.017), except for the low dose versus high dose groups (p > 0.05).

Ovariectomy and subsequent 17β-estradiol administration clearly affected the animals' body weights during the experiment. In comparison to sham-operated animals, ovariectomized rats rapidly increased in body weight, whereas the opposite was seen in rats receiving 17β-estradiol (Figure 4). In the two-way ANOVA model, all groups differed significantly (p < 0.001 to p = 0.017), except for the low dose versus high dose groups (p > 0.05).

### Food and water consumption

**Figure 5:**
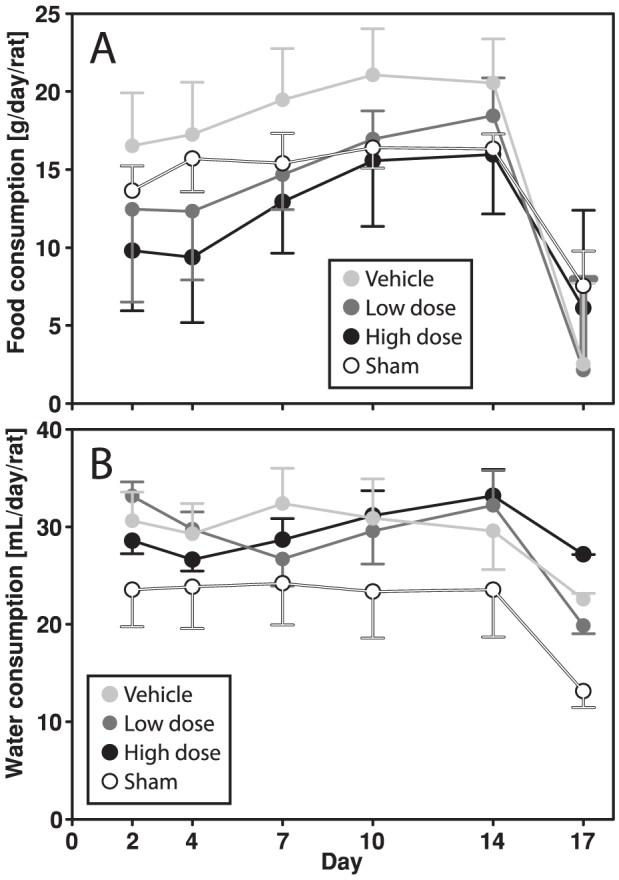
Consumption of food (A) and water (B) was significantly affected by the hormone regimens. The vehicle group ate significantly more than the low (p = 0.002) and high dose (p < 0.001) groups, while the sham group tended to drink less than the vehicle (p = 0.028) and high dose (p = 0.03) groups.

Food and water consumption were also affected by the treatments. Most notably, the weight gain in the ovariectomized rats was paralleled by an increased food intake. The vehicle group differed significantly from the low dose (p = 0.002) and high dose (p < 0.001) groups in food consumption, and the sham group differed significantly from the vehicle (p = 0.028) and high dose (p = 0.03) groups in terms of water consumption (Figures 5 A and B).

### Serum 17β-estradiol concentrations

While the low-dose hormone treatment produced hormone concentrations in the physiological range^9^, the high-dose treatment rendered levels that were at least an order of magnitude higher, which was in line with the aim of the study. Concentrations of 17β-estradiol in serum are presented in Table 3 (please note that median and inter-quartile range are used).

### Protocol violations

One animal in the vehicle group and one animal in the low dose group were excluded during MCAo due to excessive bleeding from the ICA and intracerebral hemorrhage, respectively.

### Pilot study result

In a pilot study, 12 animals were divided into three groups of four. One group received the slow-release pellet (Innovative Research of America, 90-day release, 1.5 mg) that earlier has been shown to increase ischemic damage in our lab, and the other two groups were implanted silastic capsules containing two different concentrations of 17β-estradiol (either two capsules containing a 17β-estradiol concentration of 10 000 μg/mL or two capsules containing 50 000 μg/mL). Blood samples were obtained at days 2, 7, 14 and 17, and analyzed with the radioimmunoassay described below. It was found that the highest dosed capsules produced serum concentrations of 17β-estradiol that on all occasions were higher than or equivalent to the concentrations produced by the pellets, and these were therefore chosen for the main study.

## Discussion

The main hypothesis of the study, that the high dose group would have the largest infarcts, could not be confirmed because no statistically significant differences were found between the groups. Further, because the coefficients of variations in the groups turned out to be much higher than expected, the expected power of the study was too low to support conclusions regarding similarity between groups.

Large lesion size variability is a common challenge when utilizing animal stroke models; however the literature on this specific topic is surprisingly scarce. This recently prompted us to perform a large meta-analysis to elucidate which methodological factors contribute to lesion size variability. One of the findings, although not pertaining to any of the main hypotheses of that study, was that the use of laser Doppler flowmetry seemed to lower variability^13^. Laser Doppler flowmetry is frequently used to verify proper occlusion of MCA, and animals that do not display an adequate drop in blood flow are generally excluded. The use of laser Doppler flowmetry could possibly have been an improvement to the current study by lowering infarct size variability, but we did not have access to this technology at the time. It is also worth mentioning that the use of laser Doppler flowmetry does have its disadvantages. For example, drilling a hole in the animal's skull for mounting the Doppler probe may introduce microorganisms intrathecally, which in turn could affect stroke outcome. Further, exclusion of animals using laser Doppler may mask beneficial effects of the investigated substance (in our case 17β-estradiol) on stroke mediated by changes in cerebral blood flow. Another possible improvement to the current study could have been to use rats of a different strain, since the use of Wistar rats have been demonstrated to decrease infarct size variability^13^. Sprague-Dawley is, however, extremely well-used, and the strain factor probably only contributes to a minor degree to the large variability seen in the current study^13^.

The large intra-group variability masking the effects of 17β-estradiol may be one of the explanations for the discrepancy between the current and earlier studies. The literature concerning the effects of estrogens on ischemic stroke in rats is as mentioned above, however, not very concordant. The earliest animal studies of estrogens' effects on stroke indicated beneficial effects^2^,^14^,^15^, which is also in line with the majority of later studies^8^,^16^. However, in several studies testing the effects of estrogens in similar stroke models, the hormones have on the contrary been shown to augment ischemic damage^4^,^5^,^17^. In a previous systematic analysis, we made the case that this dichotomy could be explained by discrepant estrogen administration modes, underlying differences in hormone doses delivered to the animals^11^. This hypothesis was, as mentioned above, the rationale for the current study. Several mechanisms for estrogens' protective effects in cerebral ischemia have been propagated^18^, including milder inflammatory response^19^, decreased apoptosis^20^ and reduced oxidative stress^21^,^22^. Concerning the potentially damaging effects, suggestions have been limited to pro-inflammatory effects^5^,^23^, augmented excitotoxicity^18^,^24^ and increased oxidative stress^17^,^25^. The hypothesis put forth in the current article; that the weight-loss afforded by high-dose estrogens may be damaging, has to our knowledge not been suggested earlier.

The secondary hypothesis, that the ovariectomy and subsequent 17β-estradiol regimens affected body weight and feeding behavior, was verified. A clear tendency that ovariectomy increased, while the hormone treatments decreased, feeding and body weight could be seen, which corroborates earlier studies of the effects of ovariectomy on energy balance^26^. Estrogen deficiency is known to promote feeding and weight gain, which partly can be explained by the hormones' leptin-like effects, potently regulating energy balance^27^. Concerning water consumption, all experimental groups unexpectedly seemed to increase their water intake in comparison to the sham group. The rationale for analyzing the effects of the hormone treatments on feeding behavior and body weight was that it could offer an explanation for the presumed detrimental effects of high-dose estrogens. Higher doses of estrogens should theoretically cause a more pronounced weight-loss, which in turn could augment ischemic damage, in line with the so-called “obesity paradox”. The obesity paradox states that obese individuals, although obesity in itself may have contributed the pathogenesis, are often better off than their leaner counterparts when the disease actually comes about^28^,^29^. Theoretically, this could counter-balance the protective effects of estrogens, and the net estrogenic contribution may even be made detrimental. As it turned out, with no significant effects of 17β-estradiol on lesion sizes in the current study, the eventual contribution of weight-loss to the hypothesized differences was obviously not possible to assess.

It was also noted that the ischemic stroke led to a rapid (about 10% in three days) decrease in rat body weight, irrespective of preceding hormonal status. This is probably explained by the parallel reduction in food intake by about 50% (Figure 5) in combination with the metabolic changes that accompany the extreme stress that an ischemic stroke implies^30^,^31^.

The functional tests that were applied revealed no difference between the treatment paradigms, which possibly was an effect of the large lesion size variability. However, another interesting observation was made. In the large majority of rats that were inflicted cerebral ischemia, there was a tendency of switching swing side from day 15 to day 17. On day 15 most rats swinged to the right (i.e. contralateral to the ischemic lesion), which was also the swing direction during ongoing MCAo. However, on day 17, most had switched to making left-side swings. The tail swing test, first developed for Parkinson's disease research^32^, has been used in a number of rat stroke studies^33^, and a short version of the test is also included in the Bederson^34^ and Garcia^35^ test scores. Despite its frequent use of the test and the extremely clear pattern observed in the current study, we have not been able to find any record of such a phenomenon in the previous literature. However, it requires emphasis that this type of unexpected finding should only be considered as hypothesis generating, and must be investigated further in settings specifically designed for this purpose.

In conclusion, even if the 17β-estradiol regimens clearly affected the animals' energy balance, the current study could neither corroborate nor falsify the main hypothesis due to unexpectedly large random intra-group variations. It is possible that differences would have been found had the variability been lower, and the hypothesis that high-dose estrogens may be detrimental while low-dose estrogens are protective needs to be addressed anew in an adequately modified model.

## Methods

### Animals

Seventy female Sprague Dawley rats (weight ± SEM: 281 ± 2 g; N = 70) were housed 2 in each cage (base: 21.5*36 cm, height: 18.5 cm), with nesting material (Sizzlenest, Datesand Ltd, Manchester, England), food (801730, Special Diets Service, Essex, England) and water provided *ad libitum* and 12 h/12 h light/dark cycles. In the postoperative period, the rats were housed individually and were not allowed nesting material. All procedures were conducted in accordance with the National Committee for Animal Research in Sweden and Principles of Laboratory Animal Care (NIH publication no. 86-23, revised 1985). The study was approved by the Local Ethics Committee for Animal Care and Use at Linköping University (Permit Number: 150-10).

### Experiment overview

Sixty animals were allocated individual numbers and tagged by tail coloring, after which they were randomized (Random Sequence Generator, random.org) into three groups of twenty. After administration, the animals were put in cages together with one of their group mates, and the two animals in each cage were operated on the same day to enable food and water consumption measurements. The three groups were after ovariectomy (day 0) administered vehicle (vehicle group), low dose 17β-estradiol (low dose group) and high dose of 17β-estradiol (high dose group), respectively. Another group of ten rats were sham-ovariectomized and received vehicle implants (sham group). Fourteen days after ovariectomy and vehicle/17β-estradiol administration, the rats in the vehicle, low dose and high dose groups were anesthetized and inflicted cerebral ischemia (middle cerebral artery occlusion; MCAo), while the animals in the sham group underwent a sham operation. Another three days later (day 17) all rats were sacrificed and infarct sizes analyzed (Figure 1).

#### Blinding

One of the authors, JOS, performed all experimental procedures and result analyses except for group randomizations, ovariectomies and vehicle/hormone administrations, which were performed by EI and AT. JOS was during the entire experiment blinded for the group allocations, and the treatment paradigms of the different groups were not revealed until after infarct size analyses. An exception was the sham group, since this group was only sham-ovariectomized and only underwent sham surgery instead of MCAo. Also, JOS was aware that the two animals in each cage were in the same group. Except that the two animals in each cage were operated on the same day, the rats were handled and operated on in random order.

### Surgical procedures

#### Ovariectomy

All ovariectomies were performed on day 0 via the dorsal route. The sham group underwent sham surgery, during which the ovaries were gently pulled out, but then immediately reinstated in the abdominal cavity.

#### Administration of 17β-estradiol

At day 0, right after ovariectomy, 17β-estradiol or vehicle was administered in silastic capsules, as described earlier^9^. In brief, 30 mm segments of silastic laboratory tubing (Inner/outer diameter: 1.575/3.175 mm, Dow Corning, VWR International, Buffalo Grove, IL, USA) were filled with 17β-estradiol (Sigma-Aldrich Sweden AB, CAS# 50-28-2, Stockholm, Sweden) dissolved in sesame oil (Sigma-Aldrich Sweden AB, CAS# 8008-74-0, Stockholm, Sweden), and plugged with 5 mm pieces of wooden applicator sticks (Birch, length 15 cm, diameter 2 mm, SelefaTrade AB, Spånga, Sweden). The concentrations within the capsules were 0, 180 and 50 000 μg/mL respectively. Before implantation, the capsules were stored overnight in a beaker containing sesame oil with the same concentration of 17β-estradiol as inside the capsules. During surgery, a 1 cm incision was made in the loose skin of the rat's neck, and two subcutaneous pockets was dissected caudally on each side of the rat, in which the silastic capsules were placed. All rats received two capsules, one on each side; the sham and vehicle groups received two vehicle capsules, the low dose group received one vehicle capsule and one 180 μg/mL capsule and the high dose group received two 50 000 μg/mL capsules. The high-dose regimen was in a pilot study (described below) designed to produce approximately the same serum hormone levels as did the earlier used neurotoxic slow-release pellets.

#### MCAo

The animals in the vehicle low dose and high dose groups were inflicted transient cerebral ischemia in the left hemisphere by the intraluminal filament method, based on the method described by Longa^6^, on day 14. The anesthetized animal was put in supine position, and after shaving, a 2 cm cervical midline incision was made. The common (CCA), internal (ICA) and external (ECA) carotid arteries were freed from surrounding tissue. CCA and ECA were ligated with a suture (6-0 silk suture, Johnson & Johnson, New Brunswick, NJ, USA), while ICA was temporarily clipped with a vascular microclip (8 mm artery clip, Rebstock Instruments Gmbh, Dürbheim, Germany), after which a small incision was made in the CCA. Then a 30 mm silicone coated 4-0 nylon suture (403756, Doccol, Redlands, CA, USA) was inserted and advanced up ICA approximately 18–20 mm until a mild resistance was felt, indicating correct placement. The filament was secured by a knot and the animal was allowed to wake up. After 60 minutes of occlusion, the rat was reanesthetized, the filament was withdrawn and ICA was permanently occluded. The rats were let to recover in heated (25 ± 2°C) cages for one hour, and during the first 24 h postoperatively, water-soaked food pellets were placed in a petri dish on the cage floor to promote eating. Sham rats went through identical procedures, except that after ligating CCA and ECA, the ICA was immediately ligated and the wound was closed.

#### Anesthesia and physiological monitoring

During ovariectomy, blood samplings and MCAo, the animals were anesthetized with 1.5% (4.5% for induction) isoflurane (Forene®, Abbott Scandinavia AB, Solna, Sweden) in a 30/70 mixture of O_2_/N_2_O. Body temperature was regulated by a heating pad connected to an rectal thermometer (50-7061, Harvard Apparatus, Holliston, MA, USA). Eye gel (Lubrithal™, VetXX, Uldum, Denmark) was utilized for eye protection. Five mg/kg bodyweight of carprofen (462986, Rimadyl Vet, Pfizer ApS, Ballerup, Denmark) for postoperative analgesia was administered during ovariectomy surgery, while topical lidocain gel (Xylocain 2%, AstraZeneca AB, Södertälje, Sweden) was used during MCAo. Before incisions, the skin was cleaned with Jodopax (Jodopax vet®; Pharmaxim AB, Helsingborg, Sweden). During MCAo anesthesia, saturation, breath frequency and heart rate were monitored by pulse oximetry (SLS-MO-00404, MouseOx, Allison Park, PA, USA). Physiological parameters recorded peroperatively are presented in Table 2.

### Measurement procedures

#### Food and water weighing

On days 0, 2, 4, 7, 10, 14 and 17, the rats, their food and their water were weighed, and daily consumption for each cage was calculated.

#### Blood sampling and 17β-estradiol immunoassays

On day 2, 7 and during MCAo day 14, 500 μL blood samples were collected from all animals through venipuncture (Vacuette) of the hind leg. At sacrifice on day 17, trunk blood was collected. The blood was subsequently centrifuged, the supernatant aspired and stored in −20°C until analysis.

Serum samples were analyzed by ^125^I radioimmunoassay (17β-estradiol double antibody, KE2D; Siemens Healthcare Diagnostics Inc., Tarrytown, NY, USA) and counted in a gamma counter (2470 WIZARD^2^ Automatic Gamma Counter; PerkinElmer, Waltham, MA, USA), according to a protocol that earlier has been assessed for use in rat sera^36^. Fifty μL of sample/calibrator was used for each tube, and samples were analyzed in duplicate. All used kits were of the same LOT, and reagents from different kits were pooled prior to analysis. The kit has, according to the manufacturer, a detection limit of 1.4 pg/mL and inter-/intra-assay variation of 4–14% and 3.5–5.5%, respectively, depending on the concentration range.

#### Functional tests

The rats underwent the tail swing test and cylinder test prior to surgery on day 14, and postoperatively on day 15 and 17. For the tail swing test, the animal was lifted in the tail, and the direction of the first 20 attempts to reach the experimenters hand (swings) were recorded, so that a right-left index could be calculated^37^. For the cylinder test, the rat was put in a glass beaker (diameter: 18 cm, height: 26 cm), and the 10 first times the rat reared, the use of the two paws to touch the glass was recorded, also resulting in a right-left index^38^.

#### Lesion measurement

On day 17, the rats were anesthetized in pure CO_2_ and sacrificed by rat guillotine. Brains were dissected out, immersed in ice water for one minute, and sliced in 2 mm segments in a rat brain matrix (RBM-4000, ASI Instrument Inc., USA). The slices were subsequently soaked for 20 minutes in 2,3,5-triphenyltetrazolium chloride (TTC; Sigma-Aldrich Sweden AB, CAS# 298-96-4, Stockholm, Sweden) in 0.1 mol/L PBS (pH 7.4) in a small Petri dish, maintained at 37°C in a heater. The slices were then scanned (ScanJet 2c, Hewlett-Packard Sweden AB, Solna, Sweden) and lesion sizes were analyzed similarly to the procedure described by Goldlust^39^, using an automatic 40% green spectrum threshold (SigmaScan Pro version 5, Systat Software Inc., San José, CA, USA). After infarct areas in each slice had been established, the lesion volume was calculated by multiplying the average infarct area of two adjacent slices with the thickness of the tissue in between, which then was summed up to a total infarct size. The infarct volume was corrected for edema according to the following formula:

[Infarct size] * [Total contralateral hemisphere] / [Total ipsilateral hemisphere]

### Exclusion criteria

Exclusion criteria were established prior to the start of the experiments:

- Death before the end of MCAo surgery
- Failure of implant administration, such as protrusion of capsules through the skin

### Statistics

A power analysis with an expected CV% of 40 for lesion sizes, and an expected lesion size increase of 40% in the high dose group, showed that 20 rats in each group would give a power of 0.869.

The effects of the *17β-estradiol administration regimen* on *lesion size* were analyzed by ANOVA. Lesion size data was not skewed, however, the coefficients of variation were somewhat too large to assume that data was normally distributed. Therefore, the ANOVA was complemented by “total outcome” non-parametric model according to Kruskal-Wallis, combining mortality and lesion size. Also, although not directly addressing the main hypothesis, the effect of *17β-estradiol administration regimen* on *functional tests at day 17* was analyzed by one-way ANOVA, and its effect in combination with the covariate *time* on *body weight*, *food* and *water consumption* and *serum 17β-estradiol concentrations* were analyzed by two-way ANOVA. Tukey's post-hoc test was performed on all ANOVA analyses (Systat 11, Systat Software Inc., San José, CA, USA). Significant difference with p-values < 0.05 were considered significant. Data are presented as mean ± SEM, except when presenting serum 17β-estradiol concentrations. The reason for using median and interquartile range instead was that several outliers, despite re-runs of the analysis, would have made mean ± SEM unrepresentative of the group. The outliers were probably caused by sample contamination.

### Pilot study

Before the main experiments, a pilot study was set up to design a silastic implant that would produce the same serum concentrations of 17β-estradiol as did the 1.5 mg slow-release pellets (NE-121, Innovative Research of America, Sarasota, FL, USA), that in an earlier trial have been demonstrated to increase lesion size^4^. Twelve animals were allocated to three groups of four, receiving the 1.5 mg pellets, 2 silastic capsules containing 10 000 μg/mL or 2 silastic capsules containing 50 000 μg/mL respectively. Blood samples were obtained on day 2, 7, 14 and 17, and analyzed for 17β-estradiol concentrations as described above.

### ARRIVE and STAIR

The experiment design and manuscript conform to the ARRIVE-guidelines of 2011^40^. Of the 8 STAIR-criteria^41^, developed for preclinical stroke experiments, 5 (dose-response assessment, extensive physiological monitoring, randomization and blinding, more than one effect measure, [intention to] publish in a peer-review journal) were fulfilled in current study.

## Figures and Tables

**Table 1:** Mortality

Group	N	Day 0-13	Day 14	Day 15	Day 16
Vehicle	19	0	0	2	2
Low dose	19	0	0	0	1
High dose	20	0	0	1	2
Sham	10	0	1	0	0

**Table 2:** Physiological parameters during surgery

Group	Average oxygen saturation [%]	Average pulse [bpm]	Average breath freq. [bpm]
Vehicle	98.6 ± 0.25	402 ± 5.9	50.7 ± 1.8
Low dose	98.2 ± 0.46	396 ± 7.5	46.8 ± 1.6
High dose	99.2 ± 0.1	340 ± 9.7	48.8 ± 1.7
Sham	98.7 ± 0.27	382 ± 12.3	44.6 ± 1.5

**Table 3:** Serum 17β-estradiol concentrations

Day	Median [pg/mL]	Inter-quartile range [pg/mL]
**Vehicle group**		
2	12.6	3.0–93.8
7	4.2	2.3–12.2
14	2.8	2.1–10.8
17	10.1	2.1–79.3
**Low dose group**		
2	44.3	28.2–130.2
7	17.7	10.9–27.7
14	12.7	6.8–65.8
17	18.5	6.2–49.6
**High dose group**		
2	1714.5	1590.3–1973.3
7	852.4	742.5–954.6
14	635.5	529.5–775.1
17	800.9	628.5–1335.6
**Sham group**		
2	29.5	4.9–55.4
7	4.9	3.5–947.8
14	4.6	3.0–27.2
17	2.5	2.2–4.3
